# Evaluation of the complete nuclear rDNA unit sequence of the jellyfish *Cyanea nozakii* Kishinouye (Scyphozoa: Semaeostomeae) for molecular discrimination

**DOI:** 10.1080/19768354.2021.2006309

**Published:** 2021-11-25

**Authors:** Buhari Lawan Muhammad, Yoseph Seo, Jang-Seu Ki

**Affiliations:** Department of Biotechnology, Sangmyung University, Seoul 03016, Korea

**Keywords:** *Cyanea nozakii*, jellyfish, phylogenetic analysis, tandem repeat, ribosomal DNA

## Abstract

The harmful jellyfish *Cyanea nozakii* Kishinouye has frequently occurred on Korean coasts, and its blooms have caused serious ecological and economic damages. DNA sequences of the *C. nozakii* for molecular detection and discrimination are relatively scarce. In this study, we determined the complete sequence of a single unit of tandemly repeated ribosomal DNA (rDNA) of the Korean *C. nozakii* and characterized the molecular features of the rDNA. The complete rDNA contained 8,003 bp (48.4% GC) with the same gene arrangement (18S, ITS1, 5.8S, ITS2, 28S, and IGS) to the typical eukaryotes. Dot plot analysis showed that the coding regions (18S, 5.8S, and 28S) were highly conserved, while the non-coding regions (ITS1, ITS2, and IGS) were more variable and parsimony-informative. The IGS contained a putative transcription termination signal (poly(T) tract) and four repeats of block minisatellites. Phylogenetic analyses using 18S and 28S rDNA revealed well-resolved relationships of *C. nozakii* within the order Semaeostomeae, separating it from other *Cyanea* species. The complete rDNA sequence provides various options for the selection of jellyfish taxonomic markers and may be useful for discriminating between species of *C. nozakii* and phylogeny reconstruction with close relatives.

## Introduction

The jellyfish *Cyanea nozakii* Kishinouye 1891 belongs to the family Cyaneidae and is commonly known as the ghost jellyfish. The species is widely distributed around the coasts of China, Korea, and Japan (Dong et al. 2008; Hong and Lin 2010; Liu et al. 2016). It can be industrially used as a source of collagen for immune-enhancing activity (Tang et al. 2013; Zhang et al. 2014). However, the frequent outbreaks of the jellyfish in the East China Sea and the Yellow Sea have caused serious economic and ecological problems (Ge and He 2004; Chen et al. 2005), including damage to fishing nets and preying on and killing juvenile fish, crabs and mollusks. In addition, they can produce toxins that pollute seawater and are dangerous to swimmers and marine animals (Feng et al. 2010). The causes of mass occurrences of most jellyfish (e.g. scyphozoans), however, remain poorly understood, in part due to the lack of a clear identification and phylogenetic framework for interpreting the evolution of traits linked to these events (Bayha et al. 2010).

Traditional taxonomic and morphological phylogenetic frameworks of jellyfish have been inconsistent, likely due to a relative lack of morphological characters that can reliably distinguish taxa of these invertebrates (Dawson 2003; Bayha et al. 2010). For example, within Semaeostomeae, morphology has inconsistently resolved relationships among families (Cyaneidae, Pelagiidae, and Ulmaridae), within family Pelagiidae, and within genera such as *Aurelia* and *Cyanea* (Mayer 1910; Kramp 1961; Dawson 2003). In particular, the genus *Cyanea* contains 18 species (WoRMS 2021) that are morphologically similar; specifically, the larval and juvenile stages of *C. nozakii* vary in size and shape; hence their morphological identification is difficult and time-consuming (Dong et al. 2008; Liu et al. 2016). For example, the planulae of *C. nozakii* varies from slipper-shaped to irregularly oval, measuring 90-180 mm in length and 60-95 mm in width, and the benthic scyphistomae varies in size and shape (Dong et al. 2006). In recent years, molecular investigations have reduced systematic instability by resolving inconsistencies of some morphological hypotheses at various nodes throughout the scyphozoan tree of life (Collins et al. 2006; Bayha et al. 2010; Chae et al. 2018). In addition, it offers more accurate identifications of cryptic taxa (Dawson 2003; Liu et al. 2016; Jang et al. 2020). Among the molecular methods, sequencing of ribosomal DNA is quite effective in exploring discrimination, phylogenies, and elucidating evolutionary inferences of jellyfishes (Ki et al. 2008; Chae et al. 2018).

In eukaryotes, the nuclear ribosomal DNA (rDNA) is typically structured as tandem arrays of a basic unit that contains a transcription unit which consists of 18S, ITS (ITS1. 5.8S, and ITS2), 28S, and an intervening intergenic spacer (IGS) region (Hill et al. 1990; Li et al. 2016). The rDNA clusters undergo concerted evolution and do not evolve independently from each other; however, each region of the rDNA units evolves at a different rate (Hillis and Dixon 1991). Individual rDNA regions of jellyfish have different degrees of sequence variability and varying suitability, and they can be used to answer almost any systematic questions, from studies among the basal lineages of life to relationships among closely related species and population (Li et al. 2016). The 18S and 28S genes, for example, evolve at slower rates, and they are relatively conserved and more useful for phylogenetic reconstruction of ancient evolutionary events (Field et al. 1988; Hillis and Dixon 1991), e.g. within eukaryote phyla down to the family and order levels (Passamaneck et al. 2004). In addition, highly conserved nature of the 18S and 28S rRNA gene allowed for the use of universal primers, which in general makes rRNA gene sequences easy to access (Hillis and Dixon 1991). On the other hand, the non-coding regions (ITS and IGS) evolve rapidly and have been used for reconstructing phylogenetic relationships at the species and subspecies levels, and even within species (Dai et al. 2008; Xu et al. 2016).

Previous studies had explored the phylogenetic relationships of jellyfish at higher taxonomic levels within the phylum Cnidaria, class Scyphozoa and within the order Semaeostomeae, based on 18S and 28S rDNA (Collins et al. 2006; Bayha et al. 2010; Chae et al. 2018). However, *C. nozakii* was not included in these studies probably due to the lack of available sequences of the species in public databases, which hindered clear resolution in specie-level molecular systematics of scyphozoans. In our previous study, molecular phylogeny based on mitochondrial *COI* showed that *C. nozakii* was separated from other *Cyanea* species, and the order Semaeostomeae is paraphyletic to Rhizostomeae (Karagozlu et al. 2018). However, short amplicon sequences may include less genetic variation than longer amplicons, which would reduce the ability to distinguish closely related species. To date, with the advance of molecular technology, DNA sequences of many jellyfish are available in public databases, and they are evaluated for molecular taxonomic markers. Particularly, nuclear 18S and 28S rDNA are considered the most frequently used marker for molecular taxonomy; however, only two complete nuclear ribosomal DNA belonging to *Aurelia coerulea* and *Chrysaora pacifica,* have been presented by our previous works (Ki et al. 2008; Chae et al. 2018). GenBank database only contains partial rDNA sequences (18S and ITS) of *C. nozakii* and there is no report on the complete nuclear rDNA. Providing a complete rDNA sequence of *C. nozakii* may aid in understanding the phylogenetic relationship of *C. nozakii* with other Jellyfish species and may help for its easy identification. A clear phylogenetic framework may help in interpreting the evolution of traits associated with the mass occurrence of jellyfish.

In the present study, we determined the complete nucleotide sequence of a single unit of tandemly repeated rDNA of *C. nozakii* Kishinouye and characterized the molecular features of each rDNA region. In addition, we performed comparative analyses with other jellyfish, and reconstructed a phylogenetic relationship of *C. nozakii* within the order Semaeostomeae, to gain a better understanding of the rDNA variations and evolutionary relationship of jellyfish. The data presented here may be useful for the classification, identification, and phylogenetic analyses of *C. nozakii* and other Jellyfish species.

## Material and methods

### Sampling and DNA extraction

Three specimens (RH28, RH29, and RH30) of *C. nozakii* were collected on 1 September 2015, from Tando Bay (34°58′26.4"N, 126°19′54.4"E), Korea. It is located on the west coasts of the Korean peninsula and is a closed area with weak current flow. Individual jellyfish was obtained using a hand net or a plastic bucket, and then the collected specimens were immediately fixed with 100% ethanol and transported to the laboratory. Total genomic DNA was isolated from the jellyfish umbrella using the cetyltrimethylammonium bromide (CTAB) method (Richards et al. 2003).

### PCR amplification and sequencing

Nuclear rDNA sequences were amplified by the long PCR technique with two sets of eukaryotic universal primers (forward 18F01, 5′-TATCTGGTTGATCCTGCCAGTAG-3′ and reverse 28R691, 5′-CTTGGTCCGTGTTTCAAGAC-3′; forward 28F01, 5′-CCGCTGAATTTAAGCATATAAGTAAGC-3′, reverse 18R, 5′-GCTATTGGAGCTGGAATTACC-3′). PCR reactions were carried out in 1× PCR buffer (10 mM Tris– HCl, 50 mM KCl, 1.5 mM MgCl2, 0.001% gelatin; pH 8.3) with <0.1 µg genomic DNA template, 200 µM each of the four dNTPs, 0.5 µM of each primer and 0.2 units of LA Taq polymerase (TaKaRa, Shiga, Japan). All PCR amplifications were performed in an iCycler (Bio-Rad, Hercules, CA) with the following reaction conditions: 95°C for 3 min; 35 cycles of denaturation at 95°C for 20 s, annealing at 55°C for 30 s and extension at 68°C for 5 min, and a final extension at 72°C for 10 min. The PCR products (2 µl) were analyzed by 1.0% agarose gel electrophoresis using MIDORI^Green^ (Nippon Genetics Europe GmbH, Germany) as a fluorescent source.

The confirmed samples were purified with a PCR Cleanup S & V Kit (Bionics, Daejeon, Korea). Purified PCR products were sequenced by Bionics Co., Ltd (Seoul, Korea) using the sequencing primers (Supplementary Table S1). Sequencing was performed using the BigDye™ Terminator v3.1 Cycle Sequencing Kit (Thermo Fisher Scientific), and the synthesized sequences were analyzed using an Applied Biosystems 3730xl DNA Analyzer (Applied Biosystems, Foster City, CA).

Editing and contig assembly of the rDNA sequence fragments were carried out in Sequencher v5.1 (Gene Codes Corporation, Ann Arbor, MI). The rDNA genes were identified with the help of the NCBI database and comparing with *Aurelia coerulea* (EU276014) and *Chrysaora pacifica* (KY212123) rDNA sequences. In addition, we observed typical nucleotide sequences, ‘TATCTGG’ for the start of 18S rDNA, ‘TTTGT’ for the end of 28S rRNA (Ki and Han 2005). The complete sequence of the *C. nozakii* determined here was deposited in the GenBank database (accession number MT813455).

### Sequence analyses

General molecular features (e.g. nucleotide composition, GC contents, and sequence length) of the *C. nozakii* rDNA were calculated in MEGA X (Kumar et al. 2018). In addition, GC distribution and sequence complexity across the entire rDNA nucleotides of *C. nozakii* were calculated by means of BioAnnotator in Vector NTI Advance v10.3.0 (Invitrogen, San Diego, CA). Dot plot analysis was performed by comparing the rDNA sequence of *C. nozakii* with those of the moon jelly *A. coerulea* (EU276014) and *C. pacifica* (KY212123) using MegAlign v5.01 (DNAstar Inc Madison, WI).

In addition, sequence characteristics (e.g. parsimony-informative sites, conserved sites, variable sites, and singleton) among cnidarians, including hydrozoans and scyphozoans, were determined in MEGA X using 32 and 17 sequences for 18S and 28S regions respectively, while for ITS (ITS1 5.8S, and 28S) 13 scyphozoans species were used. We used the sequences of *C. nozakii* determined in this study (MT813455), while the rest of the sequences were retrieved from GenBank database (Supplementary Table S2).

### Phylogenetic analyses

For phylogenetic analyses, we used the 18S and 28S rDNA sequences of *C. nozakii* determined in this study (MT813455). Additional sequences of its relatives were obtained from the GenBank database (Supplementary Table S2). A total of 32 and 17 sequences for 18S and 28S, respectively, were assembled and trimmed manually using Sequencher v5.1. The consensus sequences were imported into MEGA X and adjusted to the final alignment using ClustalW. The total aligned length was 1,711 bp for 18S and 1,007 bp for 28S. We performed maximum likelihood (ML), Bayesian inference (BI), and maximum parsimony (MP) analyses to infer the overall phylogenetic relationships. The ML and MP trees were constructed in MEGA X, computed with Kimura 2-parameter model (Kimura 1980) and the taxa were clustered together on 1000 bootstrap proportion (BP). The phylogenetic trees were visualized with Mega X Tree Explorer.

BI analyses were built with MrBayes v3.2.6 (Huelsenbeck and Ronquist 2001), using the GTR model with a gamma distribution for the remaining site. One million generations were run until the standard deviation of the split frequencies was < 0.01. Trees were sampled every 1000 generations, with the burning of 250 trees. The output file containing trees with posterior probabilities (PP) is shown in FigTree v1.4.4 (http://tree.bio.ed.ac.uk/).

The trees resulting from the ML analyses with BP were compared with the BI trees with PP, and MP trees with BS. The 50% majority rule consensus trees were summarized with maximum likelihood bootstrap proportion (ML-BP) and Bayesian inference posterior probability (BI-PP), and maximum parsimony bootstrap proportion (MP-BP) as nodal support. The trees were edited using Adobe Illustrator CS6 (Adobe Systems, San Jose, CA, USA).

## Results

### General features of complete rDNA unit and IGS characteristics of C. nozakii

**Figure 1.**
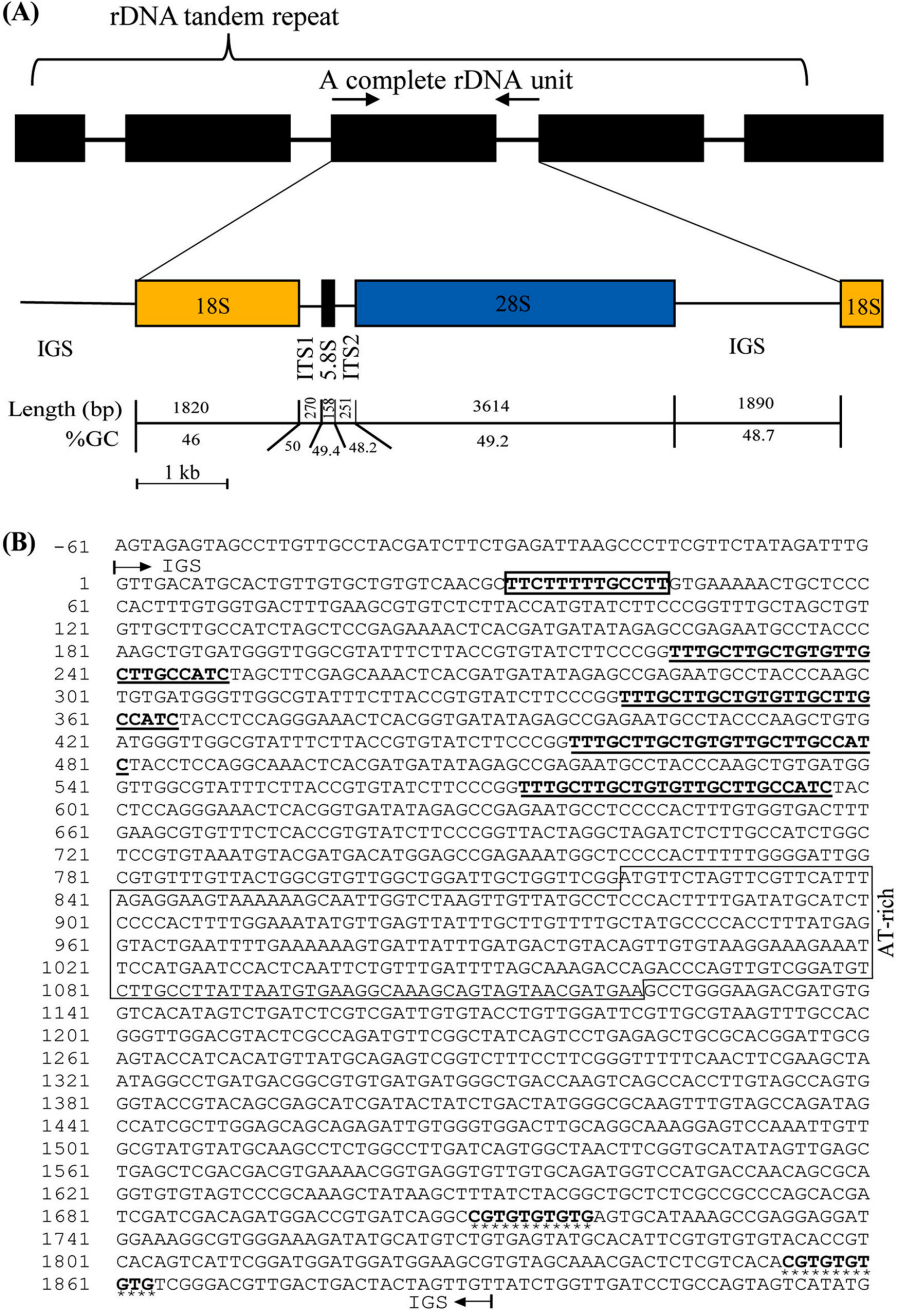
A schematic diagram of the single unit of rDNA of *Cyanea nozakii* (A). Complete nucleotide sequence of the IGS region (B). A putative termination signal (poly(T) tract) is in the solid box, and minisatellite-like nucleotides are marked by lines. The Bi-repeats are indicated by asterisks. IGS; intergenic spacer, ITS1; internal transcribe spacer 1, ITS2; internal transcribe spacer 2.

The full length of the single rDNA repeat unit of the Korean *C. nozakii* (RH28) was determined at 8,003 bp. The nucleotide frequencies of the complete rDNA were recorded at A, 25.2%; T, 26.5%; G, 27.1%; C, 21.3% (Table 1). The overall GC composition of *C. nozakii* (48.4%) was slightly higher than in *A. coerulea* (47.8%) and *C. pacifica* (46.5%). The rDNA structure of *C. nozakii* was identical to the typical rDNA of eukaryotes (Chae et al. 2018). The structure of the rDNA was organized in the following elements in order and length; 18S, 1,820 bp; ITS1, 270 bp; 5.8S, 158 bp; ITS2, 251 bp; 28S, 3,614 bp; IGS, 1,890 bp (Figure 1(A)).

**Table 1.** Characterization of the rDNA sequence of *Cyanea nozakii.*

		Nucleotide composition (%)				
Region	Length (bp)	Location	A	T	G	C
18S	1820	1-1820	26.3	27.6	26.4	19.6
ITS1	270	1821-2090	24.4	25.6	26.6	23.3
5.8S	158	2091-2248	24.7	26.0	26.6	22.8
ITS2	251	2249-2499	24.7	27.1	24.3	23.9
28S	3614	2500-6113	26.7	24.1	27.8	21.4
IGS	1890	6114-8003	21.4	29.9	26.7	22.0
Full rDNA	8003	1-8003	25.2	26.5	27.1	21.3

Upon searching the 5S rDNA database (http://combio.pl/rrna/, accessed 14 May 2021), 5S rRNA was not found in the IGS sequence of the *C. nozakii.* However, a poly(T) tract (5′-TTCTTTTGCCTT-3′) was detected at positions 32 to 45 in the IGS region at the end of 28S rDNA (Figure 1(B)). In addition, 25 bp nucleotide sequences (5′-TTTGCTTGCTGTGTTGCTTGCCATC-3′) with four repeats (block minisatellites) were identified within the IGS between the transcription termination signal and the bi-repeat sequences.

In addition, we determined additional ITS sequences of two other *C. nozakii* specimens (RH29 and RH30), and found out that they were completely identical to that of the above RH28.

### GC distribution and sequence complexity of C. nozakii rDNA

**Figure 2.**
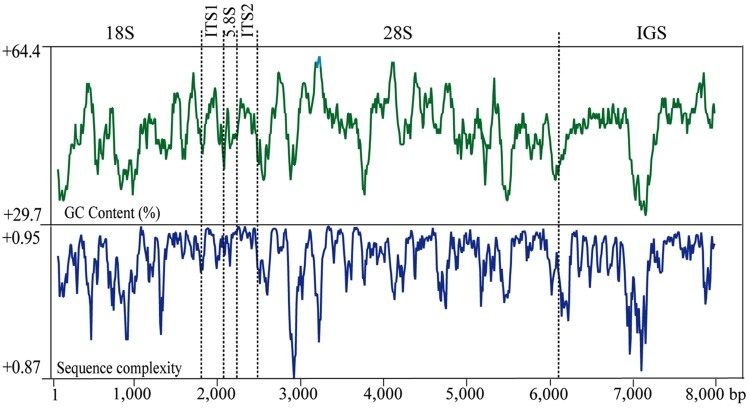
The GC content (%), sequence complexity, and entropy (dS) in 100-bp windows across the entire rDNA nucleotides of *Cyanea nozakii*.

Analyses of nucleotide distribution and sequence complexity provide an efficient way to detect simple sequence repeats and homologs patterns in the rDNA (Ki et al. 2008). Specifically, the distribution of GC content varied across the complete rDNA of *C. nozakii* (Figure 2). Among them, ITS1 has the highest GC content (49.9%), while the lowest GC content was observed in 18S rDNA (46.0%). This composition is different in *A. coerulea* rDNA (GenBank No. EU276014), in which the lowest GC content was observed in ITS1 (39.7%). However, some zones in IGS regions of *C. nozakii* showed considerably low GC content, which was similar to other jellyfish, like *A. coeruela* and *C. pacifica.* In addition, sequence variability was estimated by using sequence complexity, which presents a visualization of the complete rDNA sequence variability. The sequence complexity varied across the complete rDNA. The zone recording the lowest complexity corresponded well to the lowest GC content within the jellyfish IGS (Chae et al. 2018).

### Comparison of C. nozakii rDNA sequence and other jellyfishes

Upon comparisons of *C. nozakii* with other two available jellyfish rDNA, we found that the complete rDNA was shorter in *C. nozakii* (8,003 bp) than *C. pacifica* (8,167 bp; Chae et al. 2018), but longer than that of *A. coerulea* (7,731 bp; Ki et al. 2008). DNA similarities among the three species were measured at >97.0% in 18S, <60.4% in ITS (ITS1, 5.8S, and ITS2), >92.0% in 28S, and <29.9% in IGS.

**Figure 3.**
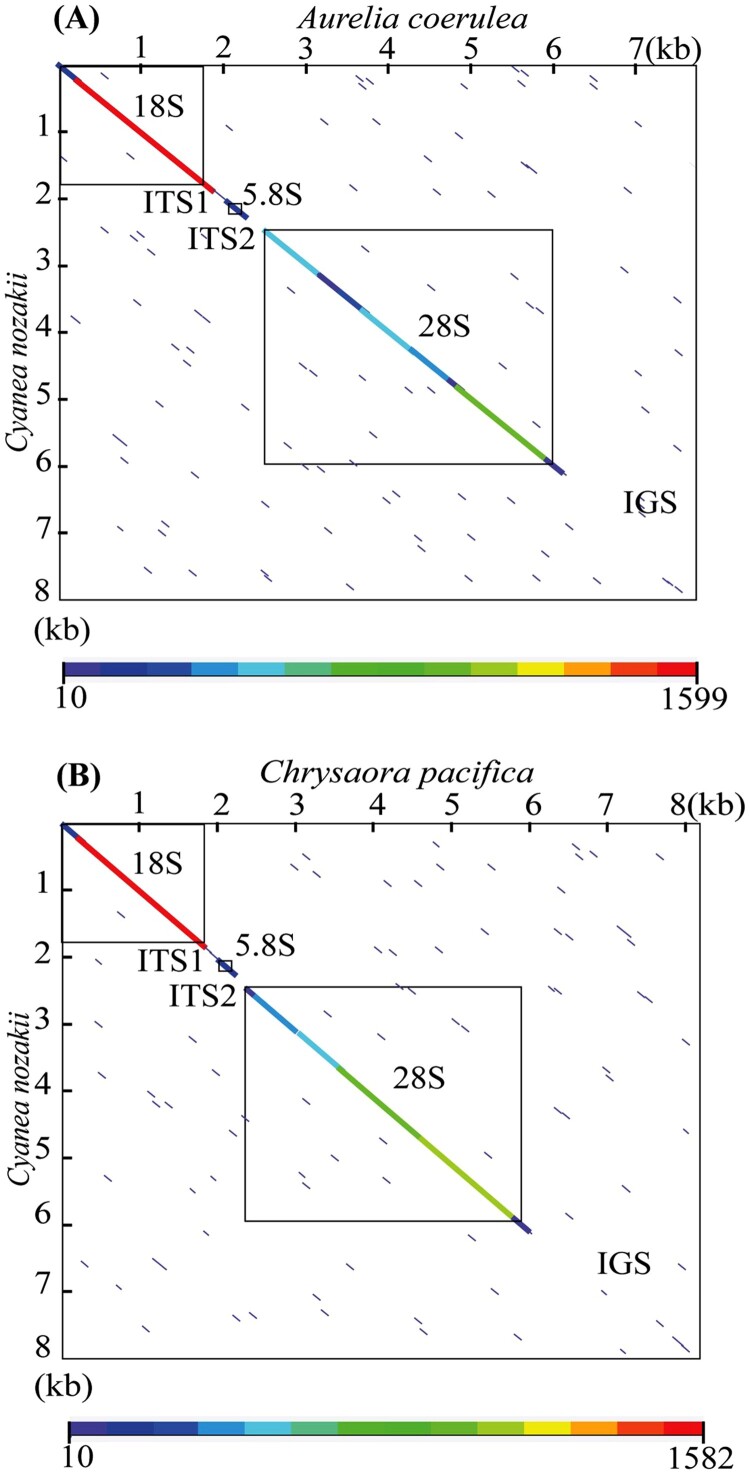
A dot plot analysis of rDNA sequences between *Cyanea nozakii* and *Aurelia coerulea* (EU276014) (A); *Cyanea nozakii* and *Chrysaora pacifica* (KY212123) (B). Color scale bars represent consecutive sequence length of some regions detected similarly between the two sequence pairs. The open boxes in matrices indicate rDNA coding regions such as 18S, 5.8S, and 28S.

Dot plots graphically revealed sequence similarities within the complete rDNA regions between *C. nozakii* and the two jellyfish; *A. coerulea* and *C. pacifica* (Figure 3). These analyses showed high similarities of the complete rDNAs at the coding regions (18S, 5.8S, 28S); however, low sequence similarities were detected in the non-coding regions (ITS1, ITS2, and IGS).

### Phylogenetic position of C. nozakii within the order semaeostomeae

**Figure 4.**
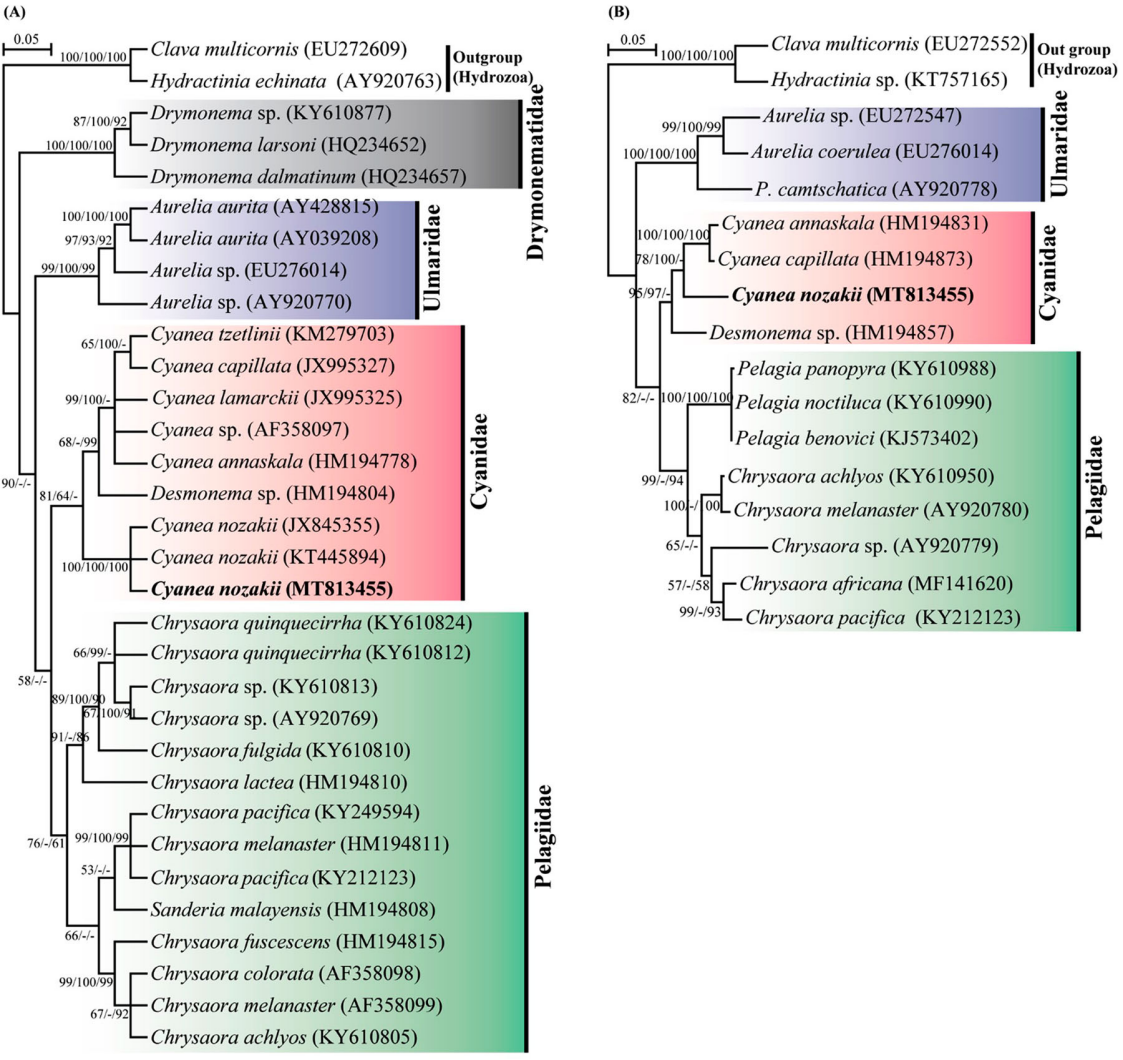
Phylogenetic relationships between *Cyanea nozakii* (MT813455) within the order Semaeostomeae inferred from nearly complete 18S rDNA (A) and partial sequence of 28S rDNA (B) with maximum-likelihood (ML), Bayesian inferences (BI) and maximum parsimony (MP). Numbers separated by a slash above each branch are; ML bootstrap probabilities (on the left side), BI posterior probabilities (in the middle), and MP bootstrap probabilities (on the right side). A nodal support with less than Branch lengths are proportional to the scale given.

In the present study, we constructed ML trees (with BI and MP support) inferred from nearly completed 18S rDNA (1711 bp) and partial sequence of 28S rDNA (1007 bp), and investigated the phylogenetic relationships of *C. nozakii* among the members of the order Semaeostomeae (Figure 4). The 18S rDNA phylogeny revealed a clear-cut separation between each family, forming separate monophyletic clades (e.g. Cyaneidae, Pelagiidae, Ulmaridae, and Drymonematidae). Cyaneidae formed a sister relationship with Pelagiidae, which was weakly supported by ML-BP (58%) but not supported by BI-PP, and MP-BP. Drymonematidae diverged the earliest in the tree (90% ML-BP; 0 BI-PP; 0 MP-BP), followed by Ulmaridae (58% ML-BP; 0 BI-PP; MP-BP). Within the order Cyaneidae, *C. nozakii* was distinct and separated from other *Cyanea* species (e.g. *C. annaskala, C. lamarckii, C. capillata and C. tzetlinii*) with moderate support (81% ML-BP; 64% BI-PP; 0 MP-BP). Additionally, 28S phylogenetic tree was congruent with the 18S analysis; Cyanidae formed a sister relationship with Pelagiidae (82% ML-BP; 0 BI-PP; 0 MP-BP), and *C. nozakii* separated from other *Cyanea* species (78% BP; 100% PP; 0 MP-BP). Parsimony-informative (PI) value constructed using the species of cnidarians and some hydrozoans revealed higher PI value in 28S (28.4%) than 18S (10.8%; Table 2).

**Table 2.** Sequence characteristics of 18S, ITS (ITS1, 5.8S, and ITS2), and 28S rDNAs among cnidarians including hydrozoans and scyphozoans.

Locus	Number of taxa	Number of sites (bp)	Conserved site (%)	Variable site (%)	Singleton site (%)	Parsimony-informative (PI) site (%)
18S	32	1711	87.5	12.4	1.6	10.8
ITS	10	417	56.0	41.0	5.5	31.8
28S	17	1007	61.5	35.6	6.9	28.4

## Discussion

Until now, over 11,000 species, including 224 Scyphozoan jellyfish, have been described in cnidarians (WoRMS 2021); however, only two entire nucleotide sequences of nuclear rDNA have been determined from the jellyfish species (Ki et al. 2008; Chae et al. 2018). In the present study, we sequence the complete nrDNA of *C. nozakii* for the first time and the results revealed that the order of genes is 18S–ITS1–5.8S–ITS2–28S–IGS, which is identical to the sequences of typical eukaryote rDNA. Upon comparisons of *C. nozakii* with two available jellyfish rDNA, we found that the complete rDNA was shorter in *C. nozakii* than *C. pacifica* (Chae et al. 2018), but longer than that of *A. coerulea* (Ki et al. 2008). The significant differences were caused by length variations in the IGS region (e.g. *C. nozakii*, 1890 bp; *C. pacifica*, 2162 bp; *A. coerulea*, 1603 bp), and are mainly attributed to differences in indels between individuals. In addition, dot plots analyses between *C. nozakii* and other two jellyfish, graphically showed that the coding regions (18S, 5.8S, 28S) have high similarities, while the non-coding regions (ITS and IGS) were highly variable. Similar results were found when comparing the complete rDNA sequence of the moon jelly *A. coerulea* with that of other invertebrates, like nematodes and insects (Ki et al. 2009).

The non-coding IGS locus contains several functional and structural elements, such as poly(T) tract, minisatellites, AT- and GC-rich sequences, and bi-repeat patterns. The poly(T) tract was observed at the beginning of the 5′ of the IGS region, which was also observed in our previous results in *A. coerulea* and *C. pacifica* (Ki et al. 2008; Chae et al. 2018), even though the sequences were not totally identical. The poly(T) tract might be used as a termination signal of the rDNA transcript (Mason et al. 1997; Ki et al. 2008) and can form hairpin structures (Ki et al. 2008). It was also observed in other marine invertebrates such as *Haliotis discus* (Guo et al. 2018) and *Perna* spp. (Guo et al. 2019), which suggest that the putative termination signal may be generally present in marine invertebrates. Moreover, we detected four repeats of block minisatellites that were also found in the IGS of other jellyfish *A. coerulea* (5′-CTAACCCTAGCCCTAACC-3′) and *C. pacifica* (5′′-TCTACTGACCACCTTTGTAAACTT-3′) with five repeats (Ki et al. 2008; Chae et al. 2018), but the sequences are completely different among the three species. These results suggest that jellyfishes may have a common minisatellite patterns in the IGS (Ki et al. 2008; Chae et al. 2018).

The rDNA genes (coding and non-coding regions) can be used as molecular markers to resolve phylogenetic relationships among cnidarians at various taxonomic levels (Ki et al. 2008; Li et al. 2016). In the present study, both 18S and 28S rDNA phylogeny revealed a clear-cut separation between each family, forming separate monophyletic clades (e.g. Cyaneidae, Pelagiidae, Ulmaridae, and Drymonematidae). At the family level, both 18S and 28S trees are not consistent with the results of our previous results (Chae et al. 2018). In Chae et al. (2018), Cyaneidae formed a sister relationship with Ulmaridae in the 18S tree, but in the 28S tree, Pelagiidae formed a clade with Ulmaridae. This discrepancy between the present study and the previous study may be caused by the lack of reference sequences of *C. nozakii* in the phylogenies of Chae et al. (2018). Because well-resolved molecular phylogenies showed that, the family Ulmaridae is a sister taxon to order Rhizostomeae rather than to Cyaneidae or Pelagiidae, thus, order Semaeostomeae is paraphyletic with respect to Rhizostomeae (Collins et al. 2006; Bayha et al. 2010; Karagozlu et al. 2018). Therefore, the present phylogenetic trees may be more accurate and better resolved than those in our previous study (Chae et al. 2018).

At the genus and inter-specie level, the phylogenetic trees in the present study are consistent with the results of previous phylogenetic studies (Collins et al. 2006; Bayha et al. 2010; Chae et al. 2018). However, *C. nozakii* was not included in the previous 18S and 28S phylogenetic trees; hence, our results revealed the first molecular taxonomic position of *C. nozakii* within the order Semaeostomeae based on rDNA genes, providing more resolved phylogenetic relationships. In the present study, both 18S and 28S regions revealed useful information and may be suitable for discriminating jellyfishes. Moreover, as judged by the PI, 28S may evolve 2.63 times faster than 18S rDNA, suggesting the 28S may be more suitable than 18S in discriminating between species of jellyfishes.

### Utilization of rDNA of *C. nozakii*

Nuclear ribosomal DNA has distinct features such as various rates of evolution among different rDNA regions, the presence of tandem repeats, and concerted evolution that occurs among repeated copies. These features are the reasons for the systematic versatility of rDNA (Hillis and Dixon 1991). Thus, regions of rDNA arrays can be used to answer almost any systematic questions and infer phylogenetic history across a very broad spectrum. The small subunit nuclear gene (18S rRNA) is the most extensively studied rRNA gene in eukaryotes and among the slowest evolving sequences found throughout living organisms; therefore, it is suitable to reconstruct ancient evolutionary events (Field et al. 1988; Hillis and Dixon 1991). On the other hand, the large subunit rRNA (28S rRNA) contains regions that evolve more rapidly than the 18S rRNA as well as regions that evolve as slowly as those in 18S rRNA, thus, can be used successfully to infer phylogenetic relationships within eukaryote phyla (Hillis and Dixon 1991; Collins et al. 2006).

The phylogenetic analyses of Bayha et al. (2010) using 18S and 28S rRNA genes is one of the most complete and statistically well-resolved molecular phylogenetic hypothesis for class Scyphozoa. The study provides the most robust framework for family-level evolutionary analyses of Scyphozoa. However, at the specie level, the phylogenetic relationships remain unresolved and in need of additional taxonomic and genomic sampling. In the present study, we provide the phylogenetic relationships using 18S and 28S rRNA genes, within the order Semaeostomeae including *C. nozakii*, providing more resolved evolutionary relationships. The phylogeny may enable the studies of evolutionary transitions in behavioral, biogeographic, ecological, and physiological traits that allow mass aggregation of some scyphozoans, including *C. nozakii* (Hamner and Dawson 2009; Bayha et al. 2010). In addition to evolutionary relationships, this study demonstrated that both 18S and 28S sequences of *C. nozakii* are unique and differentiated from other jellyfish species. The BLAST search of the 18S region showed that the *C. nozakii* in the present study was 100% identical to *C. nozakii* (KT445894) collected from Chinese coastal waters. As noted previously, *C. nozakii* is widely distributed around the coasts of China, Korea, and Japan (Dong et al. 2008; Hong and Lin 2010; Liu et al. 2016). Therefore, 18S and 28S rDNA can be utilized to easily detect and monitor this harmful organism in the marine environment. Moreover, there are no available 28S sequences of *C. nozakii* at the GenBank database, thus, the 28S sequence of *C. nozakii* reported here provides the first data for the database.

The evolutions of the ITS and IGS regions have been reported to be more rapid than that of the 18S and 28S regions (Dai et al. 2008; Xu et al. 2016). In the case of *C. nozakii*, the ITS region is more variable (40.0%) and parsimony-informative (31.1%) than 18S and 28S (Table 2). The BLAST searches of the ITS region showed a sequence similarity of 99.26% with *C. nozakii* (KR338969) and < 90% with other species. However, the BLAST searches of the IGS region did not march with any sequence from the GenBank, showing that the region was unique to *C. nozakii* determined here. The ITS and IGS can be used in further studies to explore the population differentiation of *C. nozakii* that is widely distributed around the coasts of China, Korea, and Japan. Moreover, the common minisatellite patterns found in the IGS of the three jellyfish analysed, may serve as a potential genetic marker for studies on jellyfish populations. In general, the complete rDNA sequences presented in this study can be utilized for various molecular studies of *C. nozakii* and other *Cyanea* species.

In conclusion, we reported for the first time the complete single unit sequence of the rDNA of *C. nozakii*. The total length of the ribosomal unit is 8,003 bp were arranged in the same order as that of many other eukaryotes. Phylogenetic analyses of the 18S and 28S loci revealed that the order Semaeostomeae was separated into taxonomic groups by families and genera. Even *C. nozakii* in the family Cyanidae was separated from other *Cyanea* species. The sequence presented here will enrich the GenBank database and provide significant molecular information for discriminating between species of *C. nozakii* and phylogeny reconstruction with other jellyfish.

## Supplementary Material

Supplemental Material
