# Supplementary material for: Evaluation of the complete nuclear rDNA unit sequence of the jellyfish Cyanea nozakii Kishinouye (Scyphozoa: Semaeostomeae) for molecular discrimination
Source: Anim Cells Syst (Seoul). 2021 Nov 25;25(6):424–33. doi: 10.1080/19768354.2021.2006309 (PMC8765248; doi:10.1080/19768354.2021.2006309)
Supplement: Supplemental Material [file TACS_A_2006309_SM6074.docx]

**Evaluation of the complete nuclear rDNA unit sequence of the jellyfish *Cyanea nozakii* Kishinouye (Scyphozoa: Semaeostomeae) for molecular discrimination**

**Supplementary materials**

**Supplementary** **Table S1.** Primers used in this study for PCR and sequencing (Seq.) of complete rDNA unit of *Cyanea nozakii.*

| Region | Primer | Nucleotide sequences (5′→3') | Location* | Application | Reference |
| --- | --- | --- | --- | --- | --- |
| 18S | 18F01 | CACCTGGTTGATCCTGCCAGTAG | 1–23 | PCR/Seq | This study |
|  | 18R560 | GCTATTGGAGCTGGAATTACC | 594–614 | PCR/Seq | This study |
|  | 18F800 | GCTTGAATACATGAGCATGG | 803–822 | Seq | This study |
| ITS | 18F1641 | TCCCTGCCCTTTGTACACAC | 1663–1652 | Seq | Ki and Han 2005 |
|  | 28R80 | GGGAATCCTTGTTAGTTTC | 2562–2581 | Seq | This study |
| 28S | 28F01 | CCGCTGAATTTAAGCATATAAGTAAGC | 2526–2552 | PCR/Seq | This study |
|  | 28R691 | CTTGGTCCGTGTTTCAAGAC | 3319–3338 | Seq | Ki and Han 2005 |
|  | 28F1300 | ACTGGCGATGCGGGATGAAC | 3844–3863 | Seq | This study |
|  | 28R1318 | TCGGCAGGTGAGTTGTTACAC | 3974–3994 | PCR/Seq | Ki and Han 2005 |
|  | 28F2500 | GGCGGGAGTAACTATGAC | 4968–4985 | Seq | This study |
|  | 28R2600 | TCCCTTGGCTGTGGTTTCG | 5076–5094 | Seq | This study |
|  | 28F3220 | CGTCGTGAGACAGGTTAG | 5684–5701 | Seq | This study |
| IGS | IGSR2 | CAGCCAACACGCCAGTAAC | 6901–6919 | Seq | This study |
|  | IGSR1 | TACACGCTTCCATCCATC | 7930–7947 | Seq | This study |

Note: F, forward primer; R, reverse primer; * represents location of the primers along the complete length of rDNA.

**Supplementary Table S2**. List of all the sequences retrieved from Genbank database and used in this study.

|  |  | Accession number | | |
| --- | --- | --- | --- | --- |
| S/n | Accession name | 18S | 28S | ITS |
| 1 | *Aurelia aurita* | AY428815,  AY039208 | - | AY935206 |
| 2 | *Aurelia coerulea* | - | EU276014 |  |
|  | *Aurelia limbata* | - | - | KX943300 |
| 3 | *Aurelia* sp. | EU276014,  AY920770 | EU272547 | EU276014, AY935218 |
| 4 | *Chrysaora achlyos* | KY610805 | KY610950 |  |
| 5 | *Chrysaora africana* | - | MF141620 |  |
| 6 | *Chrysaora colorata* | AF358098 | - |  |
| 7 | *Chrysaora fulgida* | KY610810 | - | MT235562 |
| 8 | *Chrysaora lactea* | HM194810 | - |  |
| 9 | *Chrysaora melanaster* | HM194811,  AF358099 | AY920780 |  |
| 10 | *Chrysaora pacifica* | KY249594,  KY212123 | KY212123 | KY212123 |
| 11 | *Chrysaora quinquecirrha* | KY610812,  KY610824 | - |  |
| 12 | *Chrysaora sp.* | KY610813,  AY920769 | AY920779 |  |
| 13 | *Clava multicornis* | EU272609 | EU272552 |  |
| 14 | *Cyanea annaskala* | HM194778 | HM194831 |  |
| 15 | *Cyanea capillata* | JX995327 | HM194873 | KM281947 |
| 16 | *Chrysaora lactea* | HM194810 | - |  |
| 17 | *Cyanea lamarckii* | JX995325 | - |  |
| 18 | *Cyanea nozakii* | KT445894,  JX845355,  MT813455 (This study) | MT813455 | KR338969, MT813455 |
| 19 | *Cyanea* sp. | AF358097 | - |  |
| 20 | *Cyanea tzetlinii* | KM279703 | - | KM281968 |
| 21 | *Desmonema* sp. | HM194804 | HM194857 |  |
| 22 | *Drymonema dalmatinum* | HQ234657 | - |  |
| 23 | *Drymonema larsoni* | HQ234652 | - |  |
| 24 | *Drymonema sp.* | KY610877 | - |  |
| 25 | *Hydractinia echinata* | AY920763 | KT757165 |  |
| 26 | *Pelagia benovici* | - | KJ573402 |  |
| 27 | *Pelagia noctiluca* | - | KY610990 |  |
| 28 | *Pelagia panopyra* | - | KY610988 |  |
| 29 | *Phacellophora camtschatica* | - | AY920778 |  |
| 30 | *Sanderia malayensis* | HM194808 | - |  |
